# Differentiated Typology of Sex Work and Implication for HIV Prevention Programs among Female Sex Workers in Nepal

**DOI:** 10.3389/fpubh.2015.00036

**Published:** 2015-03-03

**Authors:** Shiva Raj Mishra, Sanjeev Raj Neupane

**Affiliations:** ^1^Manmohan Memorial Institute of Health Sciences, Kathmandu, Nepal; ^2^Institute of Medicine, Tribhuvan University, Kathmandu, Nepal

**Keywords:** trend of sex work, condom, culture, sexual behavior, Nepal

## Abstract

**Background:** Sex work in exchange for kind and cash has long been practiced in Nepal. The HIV prevention program in Nepal is focused mainly on these two typologies of sex work. There might be more typologies of sex work beyond streets and establishments seeking research and programmatic attention. The objective of the study is to explore the differentiated typologies of sex work.

**Methods:** This is a cross-sectional study conducted using a qualitative technique. Researchers carried out eight Focus Group Discussions with female sex workers (FSWs) (*n* = 50) in different places of Tanahu district. Data were analyzed using a deductive thematic analysis approach.

**Results:** We identified a more differentiated typology of sex work. Private contact-based sex work and the covert sex work on the cruising areas along the major highways were common. Sex work has become easier to operate with the advent of new technologies such as cell phone. With limited role of facilitation by brokers and pimps, now FSWs are better off and have longer duration of relationship with clients. Soft prostitution was common, as FSWs complemented their income through sex work.

**Conclusion:** The conventional mode of peer and outreach educational approach needs to be further strengthened and modified according to the changing typology of sex work. HIV testing sites need to be further expanded to cruising areas along the highways.

## Background

Female sex workers (FSWs) are a minority group, and are marginalized socially and economically in Nepal. They harbor HIV infection and link high risk groups to low risk general population. The HIV and STI control board estimated that there are between 24,649 and 28,359 FSWs in Nepal, with an estimated 10,457 and 11,653 in Kathmandu valley alone (1). Integrated Bio-behavioral Surveillance (IBBS) 2011 reported HIV prevalence of 1.7 and 1.2% among FSWs in Kathmandu and Pokhara valley, respectively (2–4). The HIV prevalence among street-based sex workers is more than threefold higher (4.2 vs. 1.2%) (4).

There are mainly two types of sex work reported previously namely street-based sex work and the establishment-based sex work (3). The establishment-based sex work varies from sex workers as a waitress in cabin restaurants, dancers in dance restaurants, and women at massage parlors. The street-based sex workers visit cruising sites to solicit clients by them or by a broker. Street-based sex workers reported to have a high prevalence of HIV infection compared to establishment-based sex workers (3). Among the both typology of sex workers, consistent condom use with non-paying partner is relatively low. The mean number of clients served by these typology of sex work combined was 1.6 per day in Kathmandu (1.8 for street-based and 1.5 for establishment-based) (5).

The HIV prevention program in Nepal is focused mainly on these two typologies of sex work. There might be more typologies of sex work beyond streets and establishments seeking research and programmatic attention. Therefore, studying typology of sex works is important, as it can aid program managers and policy makers to focus on new typology of sex work in HIV prevention. This study has the objective to explore the differentiated typology of sex work in Nepal.

## Materials and Methods

### Study setting

This is a qualitative study. Tanahu district was selected purposively. Due to its unique location, it represents the sociopolitical and ecological scenario of two ecological regions; Terai and Hill of Nepal. This district is a semi urban, located in mid hills with estimated 348 FSWs (1.3% of national estimates of FSWs; National Center for AIDs and STD Control) (1). Tanahu has one municipality and many other semi-urban areas namely, Dulegauda, Khairenitar, Chhiran, Dumre, and Aabukhaireni (6). It is a district, which has got the longest trail of Prithvi Highway, where more than thousands of vehicles passes daily. It is a place for travel, trade, tourism, and has got many cruising areas along the highway where FSWs meet their clients in motels, lodges, and cottages.

### Data source and study techniques

Eight FGDs were carried among FSWs in four different locations of Tanahu district. A location with >50 estimated number of sex workers was selected for the study. A total of 50 FSWs participated in these FGDs. All the participants were selected purposively. We defined sex worker as any female aged 16 years and above who had been paid for sex in cash or kind in the last 12 months. Using this criterion, FSWs who give consent to participate were selected for FGD. The first author, who had relevant skills, moderated the FGDs with the use of pretested guidelines. Comments were recorded as participants responded to the facilitator and to each other. Listing of comments on newsprint in front of the room was done so as to allow participants to calibrate their thoughts with what has already been said. Discussions were carried out until the theoretical saturation was reached. Moderators recorded the discussion sessions in Nepali language. The facilitators reviewed the recorded notes and synthesized participant comments. The researchers’ goal was to arrive at themes on the topic that was shared by participants of FGDs as comments. The detail procedure for conducting a FGD is described elsewhere (7).

### Data analysis

**Figure 1:**
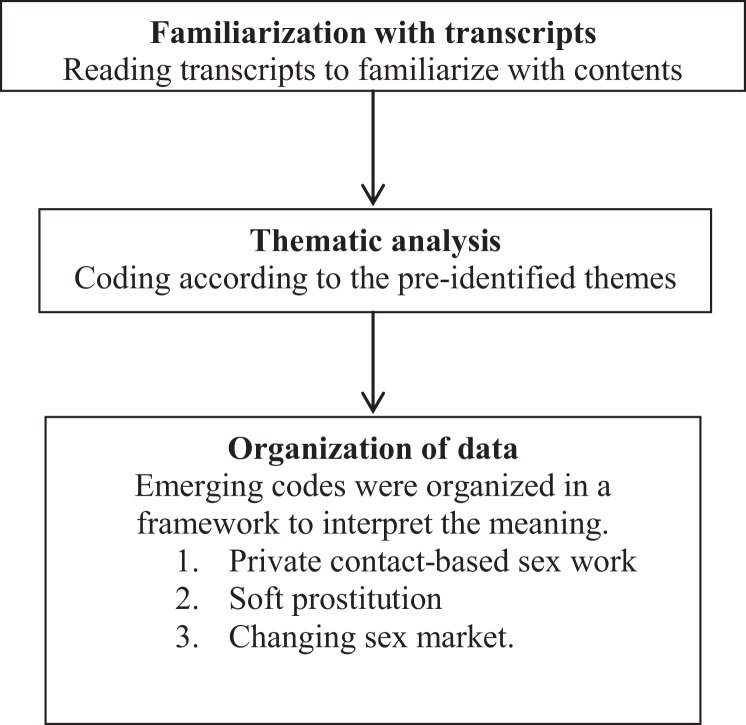
**Data analysis process**.

The recorded FGD transcripts were translated into English and back translated to Nepali by first author capable in bilingual translation. We used a qualitative deductive thematic analysis approach in analysis (8). This method has been used in Nepal by researchers (9). The translated texts were entered in MS Excel spreadsheet (10). The analysis process involved the following steps; data familiarization, data coding, and grouping into priori themes and revision. The priori themes were private contact-based sex work, soft prostitution. and changing sex market (Figure 1). These domains were developed based on authors’ own experience in the field.

### Research ethics

This research obtained ethical approval from Ethical Review Committee of Manmohan Memorial Institute of Health Sciences Kathmandu, Nepal. Prior to the interview, objectives and procedure of the research were well explained to the participants. Oral informed consent was taken from all participants before interview. Researchers did not record personal identifiers to ensure confidentiality.

## Results

### Background information about sex workers

The mean age of respondents was 28.12 years (SD = 4.51) (range of 20–35). One-fifth of them had completed secondary level of education, and one in three was illiterate. Majority of the participants in FGDs were from lower caste (30.45%) and ethnic groups (20.32%).

### Soft prostitution

Soft prostitution, this terminology has not been used previously in literature. It is about taking sex work as an income supplement, where a sex worker is partly involved in sex work and other roles in life rest of the time. They work as a laborer in agriculture and involve in household works. Sex work has been a part-time job for them. Many girls who dwell in cities and towns reported that they had involved in sex work as they have none alternatives to the sex work. They made relationship with few partners in a year for more than 6 months. Some even told that they did not like to be called as “sex worker.” Rather, they preferred to conceal their sex work. They reveal another work that they are doing side by side.

One participant said *“I didn’t need to make many partners, because I have other sources of income too. I run a boutique shop. I am making a good living”. (28 years female, Damauli)*

Another participant said *“I have relation with two partners only. One of them gives me some gifts and the other helps me with little money though I don’t ask for it. In addition to that, I work in construction work on daily wages”. (35 years female, Dulegauda)*

### Independent contact-based sex work

Majority of participants said that they need not go to hotels frequently and expect the clients. They said that they now use cell phone for communicating with their clients. According to them, they can earn better via this way rather than going to hotels directly and meeting their clients in person. On private contact-based sex, they said they did not have to share their income with hotel owners and the brokers when they contact the clients on their own. Sex workers previously worked in hotels rented rooms in towns, doing small jobs along with sex work.

One participant said *“I can earn better if I communicate with a client via cell phone than when a broker provides a client to me”. (24 years female, Dumre)*

Another participant added *“Once I started using cell phone, it has been truly easy. I need not go to hotel and risk myself of being caught by police”. (30 years female, Damauli)*

### Highway-based sex work

In highway areas, drivers and conductors had good approach with many sex workers. When they wished, they called one of sex workers of their contact to have sexual relations, on reaching the restaurants. Sometimes, hotel owners themselves assisted to find girls for them. Many of the sex workers who earlier reported worked in hotels in cities now have migrated to small motels in highways.

One participant said *“Until this year, I used to stay in Pokhara (2 hours by bus from Damauli), where there were five other girls along with me working in a hotel. Then I shifted to a highway restaurant where I am still working as a waiter. Working here is easy and the clients pay more. I have now decided to go Kathmandu. I have heard that there is better earning in Kathmandu”. (25 years female, Muglin)*

Another participant said *“My boyfriend phones me when I reach Aabukhaireni (local place near the highway). I walk from my home to a restaurant where we usually meet”. (28 years female, Aabhukhaireni)*

## Discussion

This study reported a more differentiated typology of sex work. An independent sex work outside of brothels or any such establishments reported in this study was not reported earlier in Nepal. We found that private contact-based sex work was largely fostered by the use of cell phones in sex work. Use of cell phones in client solicitation has been also reported in earlier study (11). Use of cell phones by FSWs has made the HIV prevention program harder to reach them because of their high mobility and hidden nature. Changing typology of sex work along with increased use of cell phones in client solicitation might have imposed programmatic challenges in executing HIV prevention program in Nepal. The changing scenario in typology of sex work puts the sex workers highly vulnerable to STI/HIV infections, first they cutoff from HIV program reach – access to knowledge/information about HIV and access to health care. Also, sex trade is becoming more hidden with use of cell phone. Increasing access to cell phone has greatly affected sex work in multiple ways. It has made sex work easy in operating. Now the FSWs need not be waiting in hotel or restaurants for expecting their clients to come. Firstly it has intensified the communication between the sex workers with their clients bypassing the brokers. Use of broker by FSWs to search for men (clients) and by men to search for FSWs is the usual practice in major urban areas. With the limited role of facilitation by brokers and pimps, now FSWs are better off and have longer duration of relationship with clients. Some even have one or two clients in a year. This has decreased the number of partners they could have had in a year. Having limited sexual partners is associated with lower risk of transmission of HIV (12). Though logically, visiting the same partner than with many other partners might lessen the risk of transmission of HIV. Contrarily, a study from India found use of cell phone increased HIV risk among FSWs (11). This is because of two reasons: (i) sex workers make intimate relations with their partners that they do not want to use condom and (ii) they are not able to negotiate condom use with clients outside of the places they are not familiar with (11).

National Center for AIDS and STD Control estimates, majority of sex workers, and majority of HIV infections are clustered around highway districts of Nepal (1). The speculated number of motels, lodges, restaurants in these highways cross more than thousands alone in Mahendra Highway, the longest and busiest highway in Nepal. Similar is the condition in the other highways; Prithvi highway joining Kathmandu to Pokhara, Kodari highway joining Kathmandu with Tibet, mid hill highway joining mid hills districts in Nepal. With the extending trails of highways, cruising areas along the highways might have increased. This has provided more avenues for sex workers to operate. FSWs in highway areas have the history of multiple unsafe sexual contacts with transportation workers (drivers and conductors) and with the migrants who travel from adjacent districts to abroad. The transportation workers (drivers and conductors) contact girls at cruising sites. As these workers leave the places, the girls are back to their village living the usual life. Neither majority of these sex workers have ever been tested for HIV nor do they practice safe sex (13, 14). When the clients of sex workers return to their home, they form a potential bridge of HIV infection between them and their families. This makes the low risk general population along highway districts more prone to get HIV infection.

Though the term soft prostitution is new in literature, we used soft prostitution to indicate the tendency of sex worker to use sex work as income supplement, thus reducing the number of sexual relationships that they would make. Sex workers in Nepal are engaged in variety of jobs in their life. They work as a labor in construction sites, waiter in restaurant, and beautician in parlors. In Villages, they also work in agriculture.

The current typology of sex work, mostly street-based and establishment-based might not include all the typology of sex work. An earlier research done in India recommended researchers and programmers for inclusive typology, which takes into account of HIV risk (15). In this light of findings, the more differentiated typology that we identified among FSWs needs new programmatic attention. The increasing use of cell phone can hinder HIV prevention program, as FSWs might not visit traditional venues like streets, lodges and brothels currently targeted in the program (15, 16). The HIV prevention program is now need of reorientation and restructuring so that it could reach FSWs.

This study is one of the pioneer studies to explore differentiated typologies of sex work in Nepal. Tanahu district was purposively selected for data collection. More representative survey in urban centers and highways with blended quantitative and quantitative techniques will give more generalizable findings. Further research should explore risk behaviors among different typology of sex workers and impact of cell phones in their sexual behaviors.

## Conclusion

Sex work, a stigmatized job, is an ancient practice in Nepal. We found private contact-based sex work and the covert sex work on the cruising areas along the major highways in Nepal as common. Sex work is now more private contact-based than facility based. With the advent of new technologies such as cell phones, the operation of sex work has become far easier. Now FSWs tend to have more extended relation with their clients whom they call their boyfriends, this has clearly shown the more differentiated typology of sex work in modern time. The conventional mode of peer and outreach educational approach needs to be further strengthened and modified according to the changing typology of sex work. HIV testing sites need to be further expanded to cruising areas along the highways.

## Conflict of Interest Statement

The authors declare that the research was conducted in the absence of any commercial or financial relationships that could be construed as a potential conflict of interest.
